# Factors influencing the capacity of women to voice their concerns about maternal health services in the Muanda and Bolenge Health Zones, Democratic Republic of the Congo: a multi-method study

**DOI:** 10.1186/s12913-018-2842-2

**Published:** 2018-01-25

**Authors:** Eric M. Mafuta, Tjard De Cock Buning, Didier L. Lolobi, Papy M. Mayala, Thérèse N. M. Mambu, Patrick K. Kayembe, Marjolein A. Dieleman

**Affiliations:** 1 Kinshasa School of Public Health, Faculty of Medicine, University of Kinshasa, P.O. Box 11850, Kinshasa I, Kinshasa, Democratic Republic of the Congo; 2 WOTRO Improving maternal health services performance and responsiveness through social accountability mechanisms, Athena Institute, Faculty of Life Sciences, VU University Amsterdam, Amsterdam, The Netherlands; 3 Equateur Provincial Health Division, Ministry of Public Health, Mbandaka, Democratic Republic of the Congo; 4 Kongo Central Provincial Health Division, Ministry of Health, Kimpese, Democratic Republic of the Congo; 5 Royal Tropical Institute, Amsterdam, The Netherlands

**Keywords:** Voice mechanisms, Health service responsiveness, Social accountability, DR Congo, Abuse, Disrespect, Facility delivery, Maternal mortality, Quality of care, Respectful maternal care

## Abstract

**Background:**

This paper aims to identify factors that influence the capacity of women to voice their concerns regarding maternal health services at the local level.

**Methods:**

A secondary analysis was conducted of the data from three studies carried out between 2013 and 2015 in the Democratic Republic of the Congo (DRC) in the context of a WOTRO initiative to improve maternal health services through social accountability mechanisms in the DRC. The data processing and analysis focused on data related to factors that influence the capacity of women to voice their concerns and on the characteristics of women that influence their ability to identify, and address specific problems. Data from 21 interviews and 12 focus group discussions (*n* = 92) were analysed using an inductive content analysis, and those from one household survey (*n* = 517) were summarized.

**Results:**

The women living in the rural setting were mostly farmers/fisher-women (39.7%) or worked at odd jobs (20.3%). They had not completed secondary school (94.6%). Around one-fifth was younger than 20 years old (21.9%). The majority of women could describe the health service they received but were not able to describe what they should receive as care. They had insufficient knowledge of the health services before their first visit. They were not able to explain the mandate of the health providers. The information they received concerned the types of healthcare they could receive but not the real content of those services, nor their rights and entitlements. They were unaware of their entitlements and rights. They believed that they were laypersons and therefore unable to judge health providers, but when provided with some tools such as a checklist, they reported some abusive and disrespectful treatments. However, community members asserted that the reported actions were not reprehensible acts but actions to encourage a woman and to make her understand the risk of delivery.

**Conclusions:**

Factors influencing the capacity of women to voice their concerns in DRC rural settings are mainly associated with insufficient knowledge and socio-cultural context. These findings suggest that initiatives to implement social accountability have to address community capacity-building, health providers’ responsiveness and the socio-cultural norms issues.

**Electronic supplementary material:**

The online version of this article (10.1186/s12913-018-2842-2) contains supplementary material, which is available to authorized users.

## Background

With a ratio of 846 maternal deaths per 100,000 live births in 2014 [1], the Democratic Republic of the Congo (DRC) has a high maternal mortality. Three-quarters of these deaths occur during childbirth and the postnatal period [2]. Interventions to reduce maternal morbidity and mortality emphasize facility-based childbirth and skilled attendance during delivery with timely referral for emergency obstetric care if complications occur [3, 4]. Progress towards achieving a reduction of maternal deaths has been slow because any improvements require the removal of social, financial and geographical barriers to access to skilled birth attendants, as well as addressing the health system challenges of low income countries [5–7].

To resolve this situation, additional strategies are needed beyond providing skilled personnel and improving equipment and infrastructure, such as those aiming to increase service uptake by women [8, 9]. One strategy restructures the social relationships of the main actors at stake through social accountability mechanisms. These are a set of response mechanisms that facilitate health services providers taking into consideration the needs, expectations, concerns or complaints of users about the services they provide [10, 11], and thus they improve the professional behaviour of providers towards clients [10, 11]. These voice-response mechanisms aim to make the services more responsive towards improving health service quality, contributing to an increase in health service utilization.

**Fig. 1:**
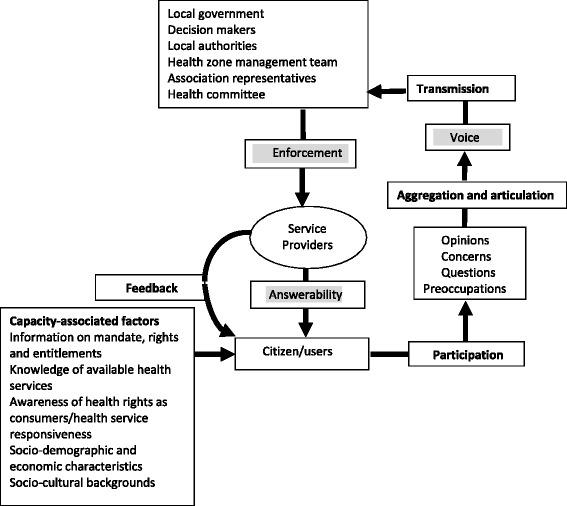
Components and steps involved in effective social accountability initiatives (adapted from Baez-Camargo and Jacobs, 2013)

Social accountability involves at least three core elements: voice, enforceability and answerability. Voice includes mechanisms, formal and informal, through which people individually or collectively express their concerns and expectations, and demand accountability from power holders. Enforceability comprises the means available to sanction non-compliance, wrongdoing and/or not appropriately fulfilling the mandate. Answerability refers to the obligation for the power holder to provide an account and the people’s right to receive a response. Social accountability also involves a feedback process through which citizens can be informed of the use made of information they have provided. To be effective, the voice of citizens needs to be articulated into actionable demands, and transmitted to the relevant actors and decision makers, who have enforcement capabilities in order to generate answerability from the service providers and local authorities (Fig. 1) [12].

In a previous study exploring existing social accountability mechanisms in rural settings in the DRC, the researchers found that very few women voiced their concerns and complaints about the health services to health providers. Interviews revealed that women in these settings are unused to expressing their concerns or expectations with the aim to improve the health service provision. Their expectations extended only to health service inputs such as assigning a doctor to the local health centre, extending the health service centre with more wards, supplying more drugs and equipment, and providing free care. They did not consider the improvement of the quality of the care provided and their own role and the role of health providers in optimizing the personal quality of care, given the financial constraints of their setting. The researchers also found that the women encountered many barriers to expressing their concerns to relevant actors and decision makers. Several factors were identified that hampered social accountability at the local level in the rural setting such as the absence of procedures to channel concerns, the fear of reprisals or of being misunderstood, as well as factors such as age-related power, ethnicity backgrounds, and women’s status [13]. Yet we do not have information on whether women in these settings are aware of their rights and entitlements, understand the mandate of the health providers, or feel abused by the health services.

According to Baez-Camargo and Jacobs, the capacity of citizens (in this case, women) to voice their concerns is influenced by the knowledge they have of the mandate of the health providers, of their rights and entitlements including their patients’ rights as consumers, and of the specific obligations that health providers have to fulfil in the course of health service provision [12]. In line with the literature on the implementation of social accountability mechanisms, the researchers assume that the capacity of these women to voice their concerns is influenced by the knowledge of their entitlements in terms of information about available maternal health services and the type, quality and quantity of care they can expect or that health providers are supposed to provide based on their mandate [14, 15] and by information about national health standards, entitlements and performance [16]. It is also influenced by their awareness about their health rights as consumers [17, 18]. In this paper, we defined the mandate of the health provider as what is expected from him/her according to the health policy [19], and the entitlement as healthcare or a health service that a woman has the right to receive from a health provider according to the health policy. The DRC national health policy, in line with the 2006 constitution, guarantees the right to health as one of the basic human rights, following international human rights treaties and the World Health Organization (WHO) constitution [7, 20, 21]. Under the 2006 constitution, it is the responsibility of national and provincial governments to protect and uphold the citizen’s rights to health services of quality-appropriate standards. Furthermore, the national health policy emphasizes communication, information and the awareness of women about the content of healthcare and advice on health as essential components that guarantee the effectiveness of maternal health [1, 7, 21]. Efforts were made towards achieving a reduction in maternal deaths by making information about a maternal healthcare package widely available to women specifically and the community in general.

To date, we have not identified a study in the DRC that has explored the factors that influence the capacity of women to voice their concerns and expectations regarding maternal health services, specifically in rural settings, in terms of knowledge of their rights and entitlements and the health providers’ mandate, and awareness of the health service they can expect to receive. This paper aims to answer the following question: What factors influence the capacity of women to voice their concerns regarding maternal health services at the rural health service level?

## Methods

### Study designs

In order to answer this research question, we re-analysed data from three studies that were previously conducted in the context of a WOTRO initiative to improve maternal health services through social accountability mechanisms in the DRC. The first study (referred to hereafter as key informant interviews) was carried out between September and October 2013 using semi-structured individual interviews. It aimed to explore the existing situation of social accountability in the two health zones [13]. The second study was a household survey combining data from two studies conducted between October and December 2014 in Bolenge Health Zone in order to identify factors associated with the use of maternal health services [22], and in Muanda Health Zone in order to analyse the effect of health providers’ responsiveness on the use of maternal health services [23]. The third study (referred to hereafter as focus group discussions) was carried out between February and May 2015. It aimed at involving community groups in designing a context-specific social accountability initiative in order to improve the performance and responsiveness of maternal health services. It used focus groups in which community groups and stakeholders were invited to discuss and reflect on findings from the previous two studies [24].

### Study settings

The three studies were carried out in two different health zones (HZs) of the 516 HZ in the DRC: the Muanda HZ in Kongo Central Province in the South-west and the Bolenge HZ in Equateur Province in the North-west. These HZs were purposefully selected because of the presence of a health partnership supporting or aiming to support an intervention involving social accountability mechanisms. Each HZ included 10 to 20 health areas. A health area is a sub-unit of a HZ covering at least 5000 inhabitants. In each HZ, one health area with a functioning health centre was randomly selected. A second health area with a functioning health centre was added for the household survey. A functioning health centre is defined according to the DRC National Health Policy as a health facility that provides an essential healthcare package at the first level, comprising basic maternal health services such as antenatal care, essential obstetric care (childbirth attended), postnatal care, family planning and tetanus immunization [21, 25].

### Data collection and issues

For the key informant interviews, used data were from 48 semi-structured, individual, audiotaped interviews conducted with an interview guide in the two selected areas: 27 in Bolenge HZ and 21 in Muanda HZ. Participants in the key informant interviews were mainly women of reproductive age (15–49 years old), expectant or with a child aged younger than six months, with or without a history of recent pregnancy complications. They were selected using purposive sampling. Other participants were women’s association representatives, female health committee members, community health workers, and women who were either mothers or mothers-in-law of a daughter or daughter-in-law who recently gave birth or was expectant. The interview guide contained questions that explored women’s experiences of the health services, women’s expectations, needs and concerns regarding maternal health services, as well as formal and informal ways to voice their concerns. A community health worker (CHW) was consulted to assist in identifying women who could be invited for an interview. Women were approached outside of their homes and invited to participate in this research. If they provided consent, the interview took place in their homes.

For the household survey, data from 517 women of reproductive age who had visited the health services for maternal healthcare were used: 195 from Muanda HZ (37.7%) and 322 (62.3%) from Bolenge HZ. They were collected through face-to-face, unique interviews in the local language using a questionnaire. Participants were selected by a multi-stage sampling procedure. In each HZ, two health areas with a functioning health centre were selected using a simple random sampling process. In each health area, all villages with more than 200 inhabitants were listed, and one-third was selected using a systematic sampling process. All households with a woman aged 15–49 years old from these selected villages who had attended a health facility for maternal healthcare, specifically for antenatal care, within the past 6 months or who had experienced childbirth in the preceding 6 months were numbered to build a sampling frame with the collaboration of the community health workers. In each health area, households satisfying the eligibility criteria were selected using a systematic sampling process, and all eligible women present in the selected households were recruited and surveyed.

The survey questions were constructed using Demographic and Health Survey (DHS) household characteristics [26], a social accountability model [27], a disrespect and abuse framework [5, 28], and the health services’ responsiveness tools [18]. The latter drew on patient health rights such as the right to be treated with respect, the right to comply or the right to an effective communication (see Additional file 1: Appendix 1). The disrespect and abuse framework was used to ensure that mistreatment was noted and measured in the same way as in recent studies. The survey was motivated by the contradictory observation that most of the women participants in the key informant interviews did not complain about the health service while other community members have asserted that there was a lot to complain about, and it aimed to assess the existence and distribution of disrespectful treatment during the use of maternal health services.

The data collection procedure was conducted as follows. After introducing herself and obtaining informed consent, the research assistant recorded the participant’s characteristics. Then the assistant told the participant two stories containing a disrespectful and abusive event and asked her whether she had experienced anything similar during her last maternal health visit (see Additional file 2: Appendix 2). In addition, the research assistant asked the respondent if she had experienced specific events, using a checklist of disrespectful and abusive events, derived from Kruk et al. (2014). This checklist was used to gain greater specificity of understanding of the woman’s experience (Additional file 1: Appendix 1). Responses to each question were categorized as ‘yes’ or ‘no’. A participant was labelled as having experienced a disrespectful and abusive event during the last health service visit if she answered ‘yes’ to at least one of the items [29]. The research assistant continued with checking the respondent’s perception of the health providers’ responsiveness and her satisfaction with the health service. Lastly, the research assistant asked the respondent whether she would visit the health service again or recommend it to her relatives based on her experiences.

For the focus group discussions, data from four focus group discussions (FGDs) held in each health area were used, amounting to a total of 92 participants. Participants in the FGDs were purposively selected among men and their community groups’ representatives, women and their community groups’ representatives, CHWs and health committee members, and key informants including health providers, the HZ management team officer, health partners and local authorities. One FGD was organized for each category in each site, and except for the latter two, the FGDs were homogeneous. Each FGD included 12 persons selected by the research team members based on a list of potential participants established by community health workers and health providers. Inclusion criteria used were: (1) aged between 17 and 75 years, (2) living in the community for more than two years, (3) belonging to the target groups.

A FGD guide was used to structure the discussion, during which the participants were invited to reflect on the results of the key informant interviews and household survey in comparison with the reality of their community. They were conducted in Lingala, and audiorecorded with the consent of the participants.

### Data processing and analysis

Transcripts from the key informant interviews and FGDs were organized and processed using Atlas-ti 7 software (ATLAS.ti GmbH, Berlin). Data processing and analysis only focused on data related to factors influencing the capacity of women to express themselves, in terms of knowledge about maternal health services and the mandate of health providers, awareness of what health services they can expect, awareness about their health rights and entitlements including their patients’ rights as consumers. The transcripts were analysed using deductive content analysis. The analysis was performed in three main stages. During the first stage, the transcripts were read repeatedly to become familiar with the participants’ stories and to identify themes associated with the ‘capacity of women’ aspects. All identified themes were recorded and labelled with a unique code to compile a list of subcategories with regard to explored aspects. During the second stage, the researchers used the list of subcategories to code each separate interview or FGD transcript. During the third stage, subcategories were merged into categories corresponding to the explored aspects by seeking connections, similarities and differences, providing a means of describing these categories and generating knowledge. The process of analysis was completed by the first author and discussed with the other authors and local health partners.

Data from the household survey recorded using Epi Info 7 (CDC, Atlanta) were analysed using SPSS 23.0 (IBM, Chicago). The data were summarized using proportions for categorical variables and means with standard deviations (SD) for quantitative variables. The association between categorical variables was tested using Pearson’s or likelihood-ratio chi-squared test as well as the Fisher test when appropriate. Proportions and means were compared using the chi-squared test and Student’s t-test, respectively. Whenever a quantitative variable was not normally distributed, the median was used for summarizing the data, and a non-parametric test was used to compare the medians. A logistic prediction model was created using the backward procedure in order to identify the characteristics of women associated with poor treatment. Independent variables included socio-demographic and health characteristics, such as age, parity, education level, marital status, occupation, religion, mode of transportation used to visit the health facility, respondent category (pregnant women or ever given birth). Other independent variables were the maternal health facility location, the fact of being informed about health facility activities, the collection of users’ views and the history of complications. The statistical significance was fixed at *p* = 0.05.

## Results

The first section describes the participants’ characteristics. The second section presents the results of factors that influence the capacity of women to express themselves, in terms of their knowledge about maternal health services and the mandate of health providers, their awareness of what health services they can expect, their ability to detect mistreatment and demand improvement, by combining data from the three studies.

### Participants’ characteristics

Since the focus of the key informant interviews was on maternal health, women of reproductive age formed the largest group. In total, 21 women of reproductive age were interviewed. Their ages ranged from 17 to 39 years (median: 27 years). The median number of children per woman was three, with ages ranging from two weeks to six years. The women were mostly farmers, with a primary school education, and lived with a partner. Participants in the household survey were 25.82 years old on average (SD = 7.34), and around one-fifth was younger than 20 years old (21.9%). The majority had not completed secondary school (94.6%) and lived with a partner (86.5%). Approximately one-third headed their household (33.3%). Most of them were farmers/fisherwomen (39.7%) or lived from odd jobs (28.2%) such as small traders, seamstresses and hairdressers. One-third of these women was pregnant (28.8%), and two-thirds had recently had a child (71.2%). Half of them already had three children. Most participants had visited the health centre in their area for antenatal care or delivery (71.2%) (Table 1). Their partners were mainly farmers/fishermen (65.4%) or lived from odd jobs (15.9%), and their level of education was mostly primary or secondary school (80.1%) (see Additional file 3: Appendix 3).

**Table 1:** Individual characteristics of respondents by abusive and disrespectful treatment, Muanda and Bolenge, 2014

Variables		Experiences of mistreatment		
	Total	Yes (*n* = 26)	No (*n* = 491)	*p*
Health zone				0.082
Muanda	195 (37.7%)	14 (7.2%)	181 (92.8%)	
Bolenge	322 (62.3%)	12 (3.7%)	310 (96.3%)	
Health catchment area				0.054*
Kitona	98 (19.0%)	5 (5.1%)	93 (94.9%)	
Nsiamfumu	97 (18.8%)	9 (9.3%)	88 (90.7%)	
Iyonda	160 (30.9%)	3 (1.9%)	157 (98.1%)	
Wendji Secli	162 (31.3%)	9 (5.6%)	153 (94.4%)	
Age				0.259
Younger than 20 years	113 (21.9%)	8 (7.1%)	105 (92.9%)	
20 years and older	404 (78.1%)	18 (4.5%)	386 (95.5%)	
Age (mean ± SD)	25.8 ± 7.3	25.4 ± 7.8	25.8 ± 7.3	0.757
Age (median, range)	24.0 (15–48)			
Education level				1.000
Below secondary school	489 (94.6%)	16 (3.3%)	462 (96.7%)	
Secondary school and above	28 (5.4%)	0 (0.0%)	28 (100.0%)	
Marital status				0.384
Live in partnership	447 (86.5%)	21 (4.7%)	426 (95.3%)	
Live out of partnership	70 (13.5%)	5 (7.1%)	65 (92.9%)	
Respondent’s occupation				0.496
Civil servant/police/army	4 (0.8%)	0 (0.0%)	4 (100.0%)	
Private sector employee	8 (1.5%)	0 (0.0%)	8 (100.0%)	
Farmer/fisherman	205 (39.7%)	9 (4.4%)	196 (95.4%)	
Small trader/odd jobs	146 (28.2%)	7 (4.8%)	139 (95.2%)	
No specific job	154 (29.8%)	10 (6.5%)	144 (93.5%)	
Respondent’s religion				0.768
Catholic	191 (36.9%)	9 (4.7%)	182 (95.3%)	
Protestant	98 (19.0%)	5 (5.1%)	93 (94.9%)	
Other Christian churches	206 (39.8%)	11 (5.3%)	189 (94.7%)	
Others (Muslim, Animist, Agnostic, Atheist)	22 (4.3%)	1 (4.5%)	21 (95.5%)	
Mode of transportation				0.536
On foot	375 (72.5%)	18 (4.8%)	357 (95.2%)	
Bicycle	44 (8.5%)	1 (2.3%)	43 (97.7%)	
Motorcycles and cars	98 (19.0%)	7 (7.1%)	91 (92.9%)	
Respondent ‘s category				0.503
Pregnant	149 (28.8%)	9 (6.0%)	140 (94.0%)	
Recently delivered	368 (71.2%)	17 (4.6%)	351 (94.4%)	
Number of deliveries (mean ± SD)	3.3 ± 2.4	3.4 ± 2.8	3.3 ± 2.4	0.786
Number of deliveries (median, range)	3.00 (0–12)	2.50 (0–12)	3.00 (0–11)	0.741
Childbirth				0.949
No previous birth and first birth	142 (27.5%)	7 (4.9%)	135 (95.1%)	
2 births and more	375 (72.5%)	19 (5.1%)	356 (94.9%)	
Health provision location				0.948
Local health area centre	368 (71.2%)	19 (5.2%)	349 (94.8%)	
Other health area facility	49 (9.5%)	2 (4.1%)	47 (95.9%)	
Health facility out of health area	100 (19.3%)	5 (5.0%)	95 (95.0%)	
Distance residence-health facility (Km) (mean ± SD)	3.2 ± 4.1	4.1 ± 6.2	3.1 ± 3.9	0.231
Informed about health centres activities (yes)	359 (62.9%)	10 (2.8%)	349 (97.2%)	0.000
Health centres collect users’ views (Yes)	288 (55.7%)	10 (3.5%)	278 (96.5%)	0.069
Complications (Yes)	97 (18.8%)	5 (5.2%)	92 (94.8%)	0.168
Ethnicity (Bantus)	499 (96.5%)	26 (5.2%)	473 (94.8%)	0.320
Native of the territory/indigenous (Yes)	255 (49.3%)	15 (5.9%)	240 (94.1%)	0.381
Knew or heard about a relative or a neighbour who experienced described situation (Yes)	38 (7.4%)	2 (7.7%)	36 (7.3%)	0.945

Participants in FGDs were aged between 22 and 67 years old. Female participants represented 42.4% (*n* = 39), and their level of education ranged from no formal education to secondary school (Table 2).

**Table 2:** Characteristics of participants in focus group discussions

Participants	Location	Number	Sex		Age	Education	
Focus groups			M	F	Age	Lower	Higher
Key informants	Muanda	12	9	3	30–65	P5	MPH
	Bolenge	8	7	1	31–45	U3	U6/MD
Community health workers and Health committee members	Muanda	12	6	6	23–67	P6	U3
	Bolenge	12	7	5	25–65	P4	U1
Men and men’s groups’ representatives	Muanda	12	12	–	25–57	P6	U2
	Bolenge	12	12	–	31–63	P4	U1
Women and women’s groups’ representatives	Muanda	12	–	12	23–45	P6	S6
	Bolenge	12	–	12	22–54	NE	S6
Total		92	53	39	22–67	NE	MPH

*Abbreviations*: *M* Male, *F* Female, *NE* No education, *P* Primary school (P6: 6th level primary school), *S* Secondary school (S2: 2nd level secondary school), *U* undergraduate (U3: 3rd level undergraduate), *MD* Medical doctor, *MPH* Master in Public Health

### Factors influencing the capacity of women to voice

Before presenting the findings on factors influencing the capacity of women to voice their views, it is worth highlighting that participants in FGDs unanimously agreed that it is important for women to express and bring forward their concerns relating to maternal health service provision to health providers. They considered that expressing their views and concerns was the only way for women to make their concerns known and to help health providers to improve the situation in case of any problem.

*“It is important to inform the nurse in charge of the health centre of our concerns…He/she needs to know in order to address this issue. If he/she does not know, this problem will continue.”*

*(Woman, FGD, Bolenge).*

On the other hand, participants in the FGDs agreed that women in local settings do not voice their concerns regarding health services. They recognized that women did not have any capacity to voice their views.

*“We do not have any capacity to speak out. We are not able to go to see health providers and to oblige them to correct this or that thing. What is the main issue? ...as it is a habit which exists...”*

*(Woman, FGD, Muanda)*

Regarding the factors that influenced the capacity of women to voice their concerns and expectations about maternal health services, data reanalysis identified four factors: (1) women’s knowledge of maternal health services and the mandate of health providers; (2) information about the health services women should expect; (3) awareness of their entitlements and rights including their rights as consumers; and (4) socio-cultural barriers to expressing themselves.

1. **Women’s knowledge of maternal health services and the mandate of health providers**

When asked to recount their experience of the maternal health services provided to them by the local health services, the majority of women faithfully described the maternal health service provision as they received it, irrespective of the type of service they received. The description of health provision by women who visited more than twice was more precise than that by those who visited once or twice. In their description, they relied on what they had received as maternal health services.

*“When we visit for antenatal care, health providers gather us together and give us a seat in a place near the health centre. The session always begins with health education and communication…They provide us with advice…Then comes the physical examination. You go into the health provider’s room, she asks you questions about your health, examines you, and takes measures of your stomach with a ribbon meter and checks it with a metal device. Sometimes, they also take your weight and direct you in the laboratories for examinations of blood, urine and stool.”(Woman, Interview, Bolenge)*

But when asked about what they knew about the health services before visiting the local health centre or about the type of health services they expected, most participants were unable to provide a clear answer.

*“Interviewer: You described what you received as healthcare during the last attendance. Did you know these before the first time? Could you talk about what you knew before you attended the service the first time?”*

*“Respondent: We did not know all these before we attended the health centre for antenatal care.”*

*(Interview, Bolenge)*

Some of them answered based on what they had heard from their mother or mother-in-law or their relatives and neighbours. Most of the time, their descriptions were based on what their sources had received themselves as maternal healthcare and not on what they had thought they should or wanted to receive as care. Those who attended antenatal care for the first time reported that they were accompanied by their mother or their mother-in-law when they lived in the same community. These companions were in charge of providing them with advice and guiding their first steps in the health services, as the interviewee lacked knowledge of the health services.Regarding the mandate of health providers related to maternal health, the majority of women answered that health providers are in charge of providing healthcare, but they were not able to explain precisely what this healthcare included and how health providers should provide it. In their account, they were unable to determine which services were missing in the health facility.

*“We are not able to understand their job. We know nothing about their work. Health providers perform their duty as they have learnt.”(Woman, FGD, Bolenge)*

The majority of women in the key informant interviews were positive about the healthcare provided to them and asserted there was nothing to complain about or request, even for non-technical aspects. Regarding their expectations and needs, the majority of women responded they had no specific expectations and needs, and they were content with the healthcare the health workers provided. They did not know what more to ask for. They believed that they were laypersons and therefore unable to judge the health providers.

*“What I want? I want that the health providers provide me with healthcare and give me necessary drugs. But I am not able to choose what care to seek for or what I need. All that health providers consider necessary for me, I accept…I am sure that they cannot harm my health.”*

*(Woman, Interview, Bolenge)*

*“We do not have a choice. All things are performed as they habitually do according to me.”*

*(Woman, Interview, Bolenge)*

(2) **Information about the health services women should expect**
  The interviews revealed that women received formal information about maternal health services through two main channels. The first channel was the health education session led by the health provider at the health centre when the women were attending maternal healthcare. The second channel was the health awareness created through home visits or mass campaigns carried out by CHWs at the household level in the community. None of the women mentioned media such as radio or the health education courses provided at school, not even the youngest participants who were still pupils. Nor did they mention information booklets and flyers, widely made available by the National Reproductive Health Programme. It also emerged from the interviews that the women received informal information about maternal health services from relatives, specifically their mother and mother-in-law, siblings, peers and neighbours. This was in line with the results of the household survey, as around seven out of ten respondents had responded that they had had some information about their health facilities and health services (69.4%) (Table 1). Half of them had been informed by health providers (50.1%), approximately a quarter by CHWs (25.3%) or neighbours or relatives (23.7%).
  However, the interviews revealed that most women who claimed to be informed about health services had information about the types of health services they could receive at the local health centre such as antenatal care, delivery, immunization or postnatal care, but not about the real content of the services they should receive (package), which seemed to be more technical information. The interviews also showed that none of these channels provided information about health service performance (health services statistics), the mandate of health providers or the rights of patients.
(3) **Awareness of their entitlements and rights**
  While it is uncommon for women specifically and people in general to know what they should expect from the technical aspects of healthcare, they are expected to be aware of non-technical aspects such as interpersonal relationships, their right to complain, and health service responsiveness. The above findings led the research team to assume that the healthcare in health settings was provided in a friendly manner, or the women were unable to detect inadequate health services, and thus not able to assert their health rights as consumers. This situation encouraged the research team to use more sensitive tools.
  The use of tools such as short illustrative stories (vignettes) and the WHO checklist aiming at improving the capture of information from the community during the household survey allowed us to detect 26 participants who reported disrespectful and abusive care while attending maternal healthcare during the survey (*n* = 571), representing 5.0% of the sample (CI 95%: 3.4–7.2%) (Table 1). The most commonly recognized event was undignified care, mentioned by ten participants. Others included inappropriate demand for payment and physical abuse. More specifically, the most common events reported were being hit/slapped/pushed/beaten, being shouted at/scolded, and being requested or receiving demands for informal payment for better care. Six participants also mentioned the experience of delivering on their own/not benefitting from antenatal services during their visit (Table 3). Furthermore, 38 participants (7.4%) asserted that they knew or heard about a relative or neighbour who experienced mistreatment like that described in the vignettes (Table 1).
  With regard to the health service responsiveness, the participants mentioned that the health providers gave them an explanation about their health problem or the healthcare they received (72.1%) (Table 4). Around half of them also asserted that the health providers listened to their opinions and views during their last visit (55.7%) and were confident that the health providers took their opinions into account (86.1%). They based their confidence on the improvement of the health services (48.8%) and good collaboration with the health providers after the feedback (12.5%).
  It emerged from the household survey that disrespectful and abusive events were mentioned by 10 participants out of 26 (38.5%) who claimed to have information about their health facilities issues, in contrast to 16 participants who did not make this claim (61.5%) (*p* < 0.001) (Table 1). Furthermore, the logistic regression performed to identify factors associated with disrespectful and abusive treatment uncovered the fact of being informed about the health facility issues as the only factor (OR = 0.245; CI 95%:0.113–0.574). The study also showed that having experienced disrespectful and abusive treatment influenced the satisfaction of the woman as user (*p* = 0.016) (Table 5). More than half of the women who experienced disrespectful and abusive events were either dissatisfied (26.9%) or neutral (26.9%). It emerged also that the proportion of women who asserted that they did not intend to attend the same health facility in the future or to recommend it to another relative was higher among those who experienced disrespectful and abusive events than among those who did not, suggesting that having experienced disrespectful and abusive events reduced the intention of visiting in the future and recommending the facility to another person (*p* < 0.001) (Table 6). The majority of women asserted that they did not have a choice of health providers as their rural area (95.6%) had a limited availability of health services (Table 4).
  The majority of participants in FGDs recognized that women were not aware of their entitlements and rights regarding the health services. It also emerged that the women were unaware that they have the right to be treated with respect and dignity or to receive the defined medical standard of the interventions and services, and that they do not have to accept some practices which are in reality abusive and disrespectful. Moreover, they asserted that they had learned from the discussion that they have the right to complain, or to be completely informed about the care they received, which they did not know before.
*“…And this is why I have already said that I cannot blame health providers, I cannot say so much as I do not know how to say if it is bad or it is done properly. For example, during the delivery, health providers slapped the woman. If she does not know if it is good or it is bad, how could she tell this doctor: you hurt me? Or the health provider acted inappropriately, but she says to herself that it is like that normally.”*
*(Woman, FGD, Muanda)*
*“If these types of meetings [FGDs] are continually organized, people will attend and gain knowledge...Then in this case, when they have to claim something from health providers, they will use clear words. They shall not doubt, the woman will not doubt either. She knows what she can say because she learned, and she knows that the thing was not done appropriately.”*
*(Man, FGD, Muanda)*
(4) **Socio-cultural barriers to expressing themselves**
  The discussion in FGDs about the extent to which the results of the key informant interviews and household survey reflected the reality of their community revealed the existence of social codes in the community under study. It emerged that, apart from CHWs and health committee members, other community member participants in the FGDs found it inappropriate to be informed about the health services’ data, as they did not work in the health services. They asserted that they did not need data or information from the health centre. They claimed that the population is not interested in learning about health centre activities because this was seen to be an attempt at controlling the health providers’ work.
*“I cannot waste my time checking the work of the health providers when I visit the health centre. It is not my job. I visit the health centre for care and not to check the others’ job”*
*(Woman, FGD, Bolenge)*
  Reacting to key informant interviews and the household survey, community members in FGDs denied the existence of disrespectful and abusive treatment at the local health centre. They asserted that they had no complaints about the health services and that they were satisfied. Regarding the complaints about physical abuse or insult that emerged from some interviews, community participants in FGDs disagreed, asserting that there was no physical abuse at their health centres, while key informants in their FGDs recognized the presence of several disrespectful and abusive treatments and promised to take action to correct the situation.
*“I think that mistreatments are very common in hospital but not here in our health centre. We have heard that in hospital, when a woman is not able to push the child out during the delivery, some birth attendants lightly slap her. It is not that she is slapped for nothing. I do not agree.”*
*(Women CHW, FGD, Muanda).*
  The community members claimed that the reports did not describe reprehensible acts but actions to encourage a woman and make her understand her situation. They asserted that the success of maternal health issues is the responsibility of the health provider, who is accountable for this, and not the woman. They recognized the action but asserted that the intention was not to harm but to encourage the woman. However, some participants stated that mistreatments are very common in the general hospital, but it is difficult to put complaints forward. Furthermore, some participants in FGDs asserted that some health providers’ reactions gave the impression of insulting women, while reprimanding women who did not adhere to the “rules”. However, they recognized it as more of a local manner of speaking rather than an insult.
*“Regarding physical abuses in the delivery room…I think that we cannot call them physical abuses or slaps. For us, there are ways to encourage women. The health providers do not slap them nor hurt them. These cannot kill them. It is to remind you that you have to make the step and push out the child.”*
*(Woman, FGD, Bolenge,)*
*“We have here women who are not able to take care of their children nor of their own hygiene. In this case, health providers act as a well-intentioned parent who reprimands her daughter. It is not a scolding nor an insult.”*
*(Woman, FGD, Bolenge).*
  Generally, the health providers, local authorities, and HZ management team officers agreed on the results presented during the meeting and on the reported abusive treatment and inappropriate behaviour of the health providers. They promised to work on improving the situation. However, they concurred that there were not many complaints about health services in local settings.

**Table 3:** Personal abusive and disrespectful experiences (n, %)

Grouped mistreatment events	Yes
Undignified care	10 (1.9%)
Shouting at patient/scolding the patient	7 (1.4%)
Threatening to withhold treatment	4 (0.8%)
Threatening comments or negative or discouraging/disparaging comments	3 (0.6%)
Abandonment or neglect	8 (1.5%)
Ignoring or abandoning patient when in need or when called	2 (0.4%)
Delivered alone/no performance of antenatal care actions during visit	6 (1.2%)
Physical abuse	8 (1.5%)
Hitting, slapping, pushing, pinching or otherwise beating the patient	8 (1.5%)
Sexual abuse or harassment	2 (0.4%)
Otherwise hurting the patient	0 (0.0%)
No/Lack of confidential care	0 (0.0%)
Allowing patient body seen by others	0 (0.0%)
Revealing confidential patient’s information to other persons	0 (0.0%)
No consented care (perform healthcare without permission/ information)	5 (1.0%)
Inappropriate demands for payment	9 (1.7%)
Request or demand for informal payment for better care	6 (1.2%)
Detention of the mother or of the baby due to failure to pay	4 (0.8%)

**Table 4:** Household survey, Assessment of health service responsiveness using adapted WHO checklist (n, %)

Health service responsiveness aspects	Yes (n = 517)
Lack of attention/health provider does not respond in reasonable time	31 (6.0%)
Health facility rooms are unclean	11 (2.1%)
Health facility rooms are small	18 (3.5%)
Did not choose the health providers	499 (95.6%)
Have an explanation of her health problem or healthcare provided	373 (72.1%)
Give her opinion in the choice of healthcare	167 (32.3%)

**Table 5:** Health service satisfaction and abusive and disrespectful experiences

Health services quality satisfaction assessment	Total	Mistreatment experiences		*p*
		Yes	No	0.016
Very satisfied	77 (15.1%)	1 (3.8%)	76 (15.7%)	
Satisfied	232 (45.4%)	11 (42.3%)	221 (45.6%)	
Indifferent/Neutral	50 (9.8%)	7 (26.9%)	43 (8.9%)	
Not satisfied/Unsatisfied	124 (24.3%)	7 (26.9%)	117 (24.1%)	
Very unsatisfied	28 (5.5%)	0 (0.0%)	28 (5.8%)

**Table 6:** Disrespectful and abusive treatment experiences and intention to utilize health services in the future

		Intention of future attendance or of recommending to another relative		
Mistreatment experiences	Total	Yes	No	0.000
Yes	26 (5.0%)	18 (69.2%)	8 (30.8%)	
No	491 (95.0%)	473 (96.3%)	18 (3.7%)	

## Discussion

While there is a growing interest in implementing social accountability mechanisms in maternal health services, there is still a need to understand the factors that influence the capacity of women to voice their concerns about health services in low- and middle-income countries. In this study, one main barrier is the knowledge gap; women at the local level have insufficient information and knowledge about health services standards and the health provider’s mandate. They are unaware of the health services they should expect according to the prevalent health policy. Furthermore, they are insufficiently informed about their entitlements and rights regarding maternal health services. Findings also show that the majority of women are poorly educated, have a low economic status, and are living in a socio-cultural context where their fundamental human rights are so frequently violated during childbirth that such care is seen by the community as normal, making them accept some categories of inappropriate care. All these elements raise barriers to detecting inappropriate care and asserting what they are entitled to. These last three characteristics also raise barriers to the women expressing themselves, even if they recognized experiences of mistreatment and inappropriate care. The study additionally shows that the use of appropriate tools such as vignettes or checklists and strategies by the research team could help women to identify some health services issues they were not aware of.

In the current study, the knowledge gap of maternal care-seeking women was evident in terms of health services standards and health providers’ mandates. This is in contrast to claims from the National Reproductive Health Programme that efforts were made to make this information widely available as an essential component in achieving the reduction of maternal mortality [7]. This knowledge gap is also described in the literature and is rooted primarily in low health literacy and the associated poor availability of health information and support, as women do not have access to sufficient information about the health services and healthcare [14, 30–32]. In most of the DRC health zones, the access to health information for the population in general and women in particular is mainly through four sources: health education courses at school; health education sessions during health visits; sensitization and health campaigns through CHWs or through the mass media; and interpersonal communication. Health education courses at school focus mostly on individual health issues and diseases rather than public health issues [33]. Other sources provide very fragmented and elementary information, which does not allow a deeper understanding of the practice of health services [34, 35]. This all contributes to knowledge asymmetry, posing a classic barrier so that when visiting the health services, a woman may not have sufficient information to judge the quality and performance, and thus may not detect inappropriate care, demand the ‘right’ kind of healthcare or assert her rights as a consumer [17, 36–38]. Thus, strategies and tools which could increase the knowledge, information or awareness of women such as WHO responsiveness [18], the disrespectful and abusive framework [5, 28, 29], media or public hearings [14] could be useful to improve the detection of inadequate health service issues [10].

According to Apolinario et al. (2013) in a Brazilian study, health literacy is also associated with socio-demographic variables, including educational attainment and major lifetime occupation. Mayuzumi (2004) found in a study in Bangladesh that some health issues also stem from deeply rooted socio-economic, cultural and environmental contexts, which people cannot easily change when operating on an individual basis [39]. In our study, most community members seemed to agree that some reported mistreatments are justified by the higher priority of delivering a healthy baby, so this act is actually a “means to encourage her” or this is related to the way of reprimanding someone using a “local manner of speaking”. This is in line with the existing literature from Tanzania [4], Ghana [40, 41] and Lebanon [42].

In an ideal world, it can be assumed that women as clients have clear knowledge about their needs when visiting a health service. They do not necessarily know what to expect from the more technical aspects of healthcare. But it can be assumed that women clearly know what they should expect in terms of interpersonal relations related to healthcare, constituting their rights e.g. to have their healthcare explained, to be informed about the disease, to receive respectful care. The study shows that women were not aware of their rights as patients. The fact that they tend not to be adequately informed of their rights nor know what to expect as healthcare suggests that they are not able to evaluate healthcare or to judge what constitutes good-quality healthcare. This situation is likely to put them in a difficult position if they wish to claim their rights. This is in line with a study from Tanzania, where the awareness of rights is considered a “new culture” [43]. Moreover, recent studies have observed that the intervention aimed at increasing women’s awareness of their rights was found to be associated with an increase in the reporting of mistreatment [44].

On the other hand, as it was assumed that women would not know what to expect of healthcare, it is the duty of health providers, in charge of providing the technical aspect of healthcare, to be more responsive during health service visits in providing them with information and explanations about healthcare. In addition, the HZ management team should supervise the health providers to make them more responsive to providing adequate healthcare, including interpersonal relation aspects.

### Study limitations

This study had some limitations related to its secondary analysis design. First, it utilizes data collected for other studies, with quite different objectives, even if the original studies aimed to understand women’s experiences of maternal health services in the DRC. The second limitation is linked to the non-equivalent and non-homogenous presence of the concepts examined by the various primary studies. The secondary analysis investigated a subject that the original analysis did not deal with. It helped to gain insights into this subject, which is important for accountability mechanisms [45]. The number of studies and the variety of methods increased the study validation by triangulation [46].

This study’s findings were limited to knowledge, information, and awareness. It did not provide insights into skills and contexts enabling women to use knowledge to voice their concerns. In addition, this study mainly addressed barriers to voicing concerns at the women’s level only, although they also exist in the power structures in the larger context. Some of these crucial aspects were investigated in previous studies [13, 24]. The study found very high levels of satisfaction among users. This situation could suggest courtesy bias, even though some authors indicated that the satisfaction with staff attitudes may not be an adequate benchmark as it is likely to be influenced by the education level and socio-cultural context [47].

### Research team and reflexivity

As with any qualitative content analysis, the data interpretation could be influenced by the background and views of the research team members. To reduce these influences, the data analysis was conducted using a framework, refining the definition of variables. Second, the materials used were taken from datasets by the researchers who collected the original research and carried out the original studies. Third, the data were collected and analysed by a research team integrating researchers from a variety of disciplines, who collaborated in the research programme with common research questions and objective. This interdisciplinarity allowed knowledge integration and limited the influence of the researcher’s subjectivity. Finally, the data were collected in interaction with the participants. The findings were discussed not only within the research team but also with local health partners, community members and health providers. The inclusion of end-users in the process allowed the integration of local knowledge and the interest of various stakeholders with different societal perspectives and who are culturally distant. This transdisciplinarity increased transparency and reliability.

## Conclusion

In summary, our findings have shown that women in rural health zones of DRC suffer mainly from a structural knowledge gap, i.e. insufficient knowledge of health services standards and the health providers’ mandate, inadequate awareness of their entitlements and rights. This is an important barrier to their voice as they are not able to detect inappropriate care and to assert what they are entitled to. In terms of socio-demographic determinants, we found that the majority of women are poorly educated, have a low economic status, and are living in a socio-cultural context that makes them accept some categories of inappropriate care as normal. Based on the findings, we suggest that initiatives to implement social accountability mechanisms must include at least a community capacity-building component in terms of basic information on healthcare standards and the health providers’ mandate and awareness of patient rights. They must also include components which address the health providers’ responsiveness in terms of improving the provision of health information during health service attendance. From our analysis, we might recommend expanding the information collection efforts to other sectors, such as integrating public health issues in school health education modules beginning in primary school and addressing some socio-cultural norms beyond providing knowledge/information.

## Additional files

Additional file 1: Appendix 1. List of patients’ rights as consumers and disrespectful items included in the checklist. (DOCX 26 kb)

Additional file 2: Appendix 2. Vignettes recounted to the participant during the survey. (DOCX 27 kb)

Additional file 3: Appendix 4. Characteristics of participants’ partners. **Appendix 5.** Households Characteristics of respondents. (DOCX 15 kb)

Additional file 4: Appendix 3. Interview guides and questionnaire used in original studies translated in English. (DOCX 41 kb)

## Abbreviations

CHWs: Community health workers
CI: Confidence Intervals
DHS: Demographic and Health Survey
DRC: Democratic Republic of the Congo
FGDs: Focus group discussions
HZs: Health zones
OR: Odds ratio
WHO: World Health Organization
