# Supplementary material for: Factors influencing the capacity of women to voice their concerns about maternal health services in the Muanda and Bolenge Health Zones, Democratic Republic of the Congo: a multi-method study
Source: BMC Health Serv Res. 2018 Jan 25;18:37. doi: 10.1186/s12913-018-2842-2 (PMC5784705; doi:10.1186/s12913-018-2842-2)
Supplement: Supplementary file 1 — List of patients’ rights as consumers and disrespectful items included in the checklist. (DOCX 26 kb) [file 12913_2018_2842_MOESM1_ESM.docx]

Appendix 1. List of patients’ rights as consumers and disrespectful items included in the checklist.

| Part 1. Patient rights as consumers |
| --- |
| - Right to be treated with respect; - Right to be free of any discrimination and exploitation; - Right to dignity, independence and autonomy; - Right to an effective communication; - Right to be fully informed; - Right to health services of appropriate standards; - Right to grant informed consent; - Right to complain; - Right to confidentiality. |
| Part 2. Disrespect and abuse items included in the checklist |
| - Body seen by others; - Shouting/scolding; - Request or suggestion of bribes or informal payments for better care; - Threatening to withhold treatment; - Threatening comments or negative or discouraging/disparaging comments; - Ignoring or abandoning patient when in need; - Delivered alone; - Non-consent for tubal ligation; - Non-consent for hysterectomy; - Non-consent for Caesarean section; - Hitting, slapping, pushing, pinching or otherwise beating the patient; sexual harassment; - Rape and detention due to failure to pay. |
