# Supplementary material for: Factors influencing the capacity of women to voice their concerns about maternal health services in the Muanda and Bolenge Health Zones, Democratic Republic of the Congo: a multi-method study
Source: BMC Health Serv Res. 2018 Jan 25;18:37. doi: 10.1186/s12913-018-2842-2 (PMC5784705; doi:10.1186/s12913-018-2842-2)
Supplement: Supplementary file 2 — Vignettes recounted to the participant during the survey. (DOCX 27 kb) [file 12913_2018_2842_MOESM2_ESM.docx]

Appendix 2. Vignettes recounted to the participant during the survey

| Dear participant,  I will recount to you two short stories about two ladies living in another village. The first story is about a lady named Marie. This lady was seven months' pregnant and went to the local health centre for antenatal care. Although she reached the health centre on time, the nurse made her wait on a bench for more than four hours before attending to her. While attending to her, the nurse left the door open, allowing other patients to see her body and Marie was asked for extra money at the end of the examination.  I would like to know whether you encountered a similar situation yourself during your last visit to the local health centre for antenatal care. Have you ever encountered another situation that you were unhappy with?  The second story is about another lady named Anne who, for her second pregnancy, visited the local health centre for the delivery. As she did not appropriately follow the antenatal care schedule, the midwife scolded her in front of other women and did not assist her when her labour started, and slapped her on the thigh because she was unable to push the baby out.  I would like to know whether you encountered a similar situation yourself during your last visit to the local health centre for delivery. Have you ever encountered another situation that you were unhappy with? |
| --- |
