# Supplementary material for: Factors influencing the capacity of women to voice their concerns about maternal health services in the Muanda and Bolenge Health Zones, Democratic Republic of the Congo: a multi-method study
Source: BMC Health Serv Res. 2018 Jan 25;18:37. doi: 10.1186/s12913-018-2842-2 (PMC5784705; doi:10.1186/s12913-018-2842-2)
Supplement: Supplementary file 3 — Characteristics of participants’ partners. Appendix 5. Households Characteristics of respondents. (DOCX 15 kb) [file 12913_2018_2842_MOESM3_ESM.docx]

Appendix 4. Households Characteristics of respondents by mistreatment, Muanda and Bolenge, 2014.

|  |  | Experiences of mistreatment | |  |
| --- | --- | --- | --- | --- |
|  | Total | Yes (n=26) | No (n=491) | p |
| Household head |  |  |  |  |
| Sex |  |  |  | 0.561 |
| Male | 456 (88.2%) | 22 (4.8%) | 434 (95.2%) |  |
| Female | 61 (11.8%) | 4 (6.6%) | 57 (93.4%) |  |
| Age (mean ± SD) | 39.3 ± 11.5 | 39.5 ± 13.0 | 39.3 ± 11.4 | 0.900 |
| Age (median) | 38.0 (18-84) |  |  |  |
| Religion |  |  |  | 0.777** |
| Catholic | 197 (38.1%) | 10 (5.1%) | 187 (94.9%) |  |
| Protestant | 99 (19.1%) | 5 (5.1%) | 94 (94.9%) |  |
| Kimbanguist | 21 (4.1%) | 0 (0.0%) | 21 (100.0%) |  |
| Muslim | 26 (5.0%) | 2 (7.7%) | 24 (92.3%) |  |
| Evangelical churches | 123 (23.8%) | 3 (2.4%) | 120 (97.6%) |  |
| Jehovah’s witness | 9 (1.7%) | 1 (11.1%) | 8 (88.9%) |  |
| Other independent Christians | 17 (3.3%) | 3 (17.6%) | 14 (82.4%) |  |
| Animist | 3 (0.6%) | 0 (0.0%) | 3 (100.0%) |  |
| Branhamist | 7 (1.4%) | 1 (14.3%) | 6 (85.7%) |  |
| No religion | 15 (2.9%) | 1 (6.7%) | 14 (93.3%) |  |
| Occupation |  |  |  | 0.437** |
| Civil servant/police/army | 38 (7.4%) | 2 (5.3%) | 36 (94.7%) |  |
| Private sector employee | 26 (5.0%) | 0 (0.0%) | 26 (100.0%) |  |
| Farmer/fisherman | 338 (65.4%) | 18 (5.3%) | 320 (94.7%) |  |
| Small traders | 26 (5.0%) | 1 (3.8%) | 25 (96.2%) |  |
| Other small jobs | 82 (15.9%) | 5 (6.1%) | 77 (93.9%) |  |
| Jobless | 7 (1.4%) | 0 (0.0%) | 7 (100.0%) |  |
|  |  |  |  |  |
| Education level |  |  |  | 0.054 |
| Lower to secondary school | 414 (80.1%) | 17 (4.1%) | 397 (95.9%) |  |
| Secondary education and greater | 103 (19.9%) | 9 (8.7%) | 94 (91.3%) |  |
|  |  |  |  |  |
| Household size (mean±SD) | 6.5 ± 3.0 | 6.3 ± 2.5 | 6.5 ± 3.0 | 0.655 |

Appendix 5. Characteristics of respondents’ partners or husband by mistreatment experiences, Muanda and Bolenge, 2014.

|  |  | Mistreatment experiences | |  |
| --- | --- | --- | --- | --- |
|  | Total | Yes (n=26) | No (n=491) | p |
| Same as household head | 360 (67.7%) |  |  |  |
|  |  |  |  |  |
| Occupation |  |  |  | 0.364** |
| Civil servant/police/army | 29 (5.6%) | 2 (6.9%) | 27 (93.1%) |  |
| Private sector employee | 27 (5.2%) | 0 (0.0%) | 27 (100.0%) |  |
| Farmer/fisherman | 318 (61.5%) | 14 (4.4%) | 304 (95.6%) |  |
| Small traders | 31 (6.0%) | 2 (6.5%) | 29 (93.5%) |  |
| Other small jobs | 91 (17.6%) | 7 (7.7%) | 84 (92.3%) |  |
| Jobless | 13 (2.5%) | 0 (0.0%) | 13 (100.0%) |  |
| Pupil/Student | 8 (1.5%) | 1 (12.5%) | 7 (87.5%) |  |
|  |  |  |  |  |
| Age of partner |  |  |  | 0.805 |
| Less than 20 years | 13 (2.5%) | 1 (7.7%) | 12 (92.3%) |  |
| 20-24 years | 83 (16.1%) | 5 (6.0%) | 78 (94.0%) |  |
| 25 years and more | 421 (81.4%) | 20 (4.8%) | 401 (95.2%) |  |
| Age (mean±SD) | 32.6 ± 8.7 | 32.1±9.7 | 32.6 ±8.6 | 0.766 |
| Age (median, range) | 32.0 (16-68) |  |  |  |
|  |  |  |  |  |
| Religion |  |  |  | 0.443** |
| Catholics | 183 (35.4%) | 8 (4.4%) | 175 (95.6%) |  |
| Protestants | 103 (19.9%) | 5 (4.9%) | 98 (95.1%) |  |
| Kimbanguists | 18 (3.5%) | 0 (0.0%) | 18 (100.0%) |  |
| Muslims | 25 (4.8%) | 2 (8.0%) | 23 (92.0%) |  |
| Evangelical churches | 131 (25.3%) | 6 (4.6%) | 125 (95.4%) |  |
| Jehovah witness | 10 (1.9%) | 1 (10.0%) | 9 (90.0%) |  |
| Other independent Christians | 19 (3.7%) | 3 (15.8%) | 16 (84.2%) |  |
| Animists | 6 (1.2%) | 0 (0.0%) | 6 (100.0%) |  |
| No religion | 15 (2.9%) | 0 (0.0%) | 15 (100.0%) |  |
| Branhamists | 7 (1.4%) | 1 (14.3%) | 6 (85.7%) |  |
|  |  |  |  |  |
| Level of education |  |  |  | 0.272 |
| Less than secondary school | 403 (77.9%) | 18 (4.5%) | 385 (95.5%) |  |
| Secondary school and up education | 114 (22.1%) | 8 (7.0%) | 106 (93.0%) |  |
