# Supplementary material for: Factors influencing the capacity of women to voice their concerns about maternal health services in the Muanda and Bolenge Health Zones, Democratic Republic of the Congo: a multi-method study
Source: BMC Health Serv Res. 2018 Jan 25;18:37. doi: 10.1186/s12913-018-2842-2 (PMC5784705; doi:10.1186/s12913-018-2842-2)
Supplement: Supplementary file 4 — Interview guides and questionnaire used in original studies translated in English. (DOCX 41 kb) [file 12913_2018_2842_MOESM4_ESM.docx]

## Appendix 3: Interview guide for with women users and no users

**Study Title: Improving performance and responsiveness of maternal health services through political accountability mechanisms in DR Congo.**

**Research Supervisors: Mambu Nyangi Thérèse, Dieleman Marjolein**

**Student Researcher: Mafuta Eric**

**Performance Sites: Muanda/Bas Congo, Bolenge/Equateur**

**Sponsor: WOTRO program/VU University Amsterdam**

| N° | Themes | Code |
| --- | --- | --- |
|  | INTRODUCTION |  |
|  | We would like first to talk about your experiences with health care during pregnancy or not but related to mother health.   - What type of services do you use as mother or pregnant woman? |  |
|  | PERFORMANCE OF HEALTH SERVICE |  |
|  | We want to talk about the encounter between the health provider and you during maternal health service attendance in your local health facility:   - Can you talk about your last encounter with the health provider especially about the way you are treated from the moment you arrived to the moment you left *( communication, attention to your word, provider’s behavior during that encounter, Discretion, confidence, Sincerity, Mutual respect)* ? - What did you think about this encounter? Was it positive or negative?   *( confidence, trust, place, process, hygiene, materials, medicines, schedule)* |  |
|  | We will talk now about the perception of the quality of maternal health services:   - What do you think make the quality of health services? - Do you think that you received quality health care during this last encounter? *(schedule, effectiveness, distance, cost, beliefs and values, availability, flexibility, agreement, acceptability)* - During your encounter, do you have the impression to be free to choose what service or good you need? Or do you feel pressed by a health provider? - Have you known before attending what care you will receive? - Are you ready to return to attend this health provider or will you be ready to recommend the local health provider to your friend or to your relative? |  |
|  | DELEGATION |  |
|  | - What are your expectations, needs, desires from health care providers? - Did the health services provided meet your expectation? How (yes) and why (no)? - What are your ideas how health care should be provided? *What services do you think must be provided? What services do you think are not provided or are not provided in the way you think it should be provided?* |  |
|  | RESOURCES SUPPLY/FINANCING |  |
|  | You talked about health services you can obtain from health providers.   - Do you think that health providers have resources (technical, financial, materials…) to accomplish their duties, responsibilities or in other words what you need them to do ? if yes, how? If not why? |  |
|  | INFORMATION |  |
|  | We will talk now about the way information from clients and users reach health services providers:   - How do you let health providers know your needs, questions, expectations, opinion or concerns? How do you think this can reach them (systems, mechanism, procedures) ?   *(No feedback from clients, suggestions box, available questionnaire for clients, users’ survey, Official meeting with community leaders, informal discussion with clients or community, protest, feedback, demonstration, coalition, consumers league…..)*   - Do you think that there are persons, individual or groups or organizations that could facilitate you to transmit your view to providers? |  |
|  | RESPONSIVENESS |  |
|  | We will talk about what is done by the health providers to address your need or concerns:   - Did you perceive some changes made by health providers according to the ideas, needs, expectations or concerns expressed by patients? If Yes, Could you indicate what changes were made following what health service area (services, schedule, operations, comfort,)? - Do you think that health providers value yours views? - Do you feel that it possible for you or the population as a group to ask questions, or to express its concerns, to obtain answers? If yes, how? If no why? |  |
|  | ENFORCEABILITY |  |
|  | We will talk about mechanism or process which can allow clients or their delegate to reinforce or to discourage providers’ behaviors:   - Do you think that health providers are responsible for their behavior; actions and results? - How do you think that you can influence the performance and the behavior of health providers? - What sanctions or penalties do you know to be applied to health providers? *Do you think that there are procedures or mechanisms set up for encouraging good performance or for discouraging the worst ones among health providers?* - Do you know individual or groups or organizations that can allow clients or users to hold health providers responsible *or to make them justify the actions, results or behaviors in front of the community?* |  |
|  |  |  |
|  |  |  |
|  | CONTEXT |  |
|  | We have discussed about mechanisms or process which can allow clients or the representative to hold health providers responsible or accountable for results, actions or behaviors.   - Do you think that there are social or political or local situation or elements from politics, society or others which can make these mechanisms work or not? *Do you think that the context (politics, society, economy or local situation) influence the behavior or the actions of health providers?* |  |
|  | MECHANISMS |  |
|  | We were talking about changes made by health providers according to the ideas or the needs of users. We need to understand why these can occur or why these can not occur ?   - What motivate health providers to meet users’ needs or demands? - What way does voice intervention may work to improve health service delivery? To improve health service uptake by the population? |  |

**KINSHASA SCHOOL OF PUBLIC HEALTH**

**WOTRO IMPROVING MATERNAL HEALTH THROUGH SOCIAL ACCOUNTABILITY IN DRC**

**INTERVIEW GUIDE WITH KEYS INFORMANTS**

**HEALTH ZONE: ……………………… HEALTH AREA: ……………………… DATE: ………………………………………**

**A. Introduction**

The Government of the Democratic Republic of the Congo gets support from other governments from other countries and organizations to fund projects in the health sector. All these projects aim at improving health situation of the population and ensuring that the population have access to quality and comprehensive health care and effectively use the available health services

Among his missions, the Kinshasa School of Public Health aims at supporting the Ministry of Public Health by collecting data in health facilities and communities in order to provide relevant information for decision making. This interview is set in this context. It is organized to discuss with representatives of the community and other stakeholders about health problems, support they perceived and solutions provided.

**B. Informed Consent process *(to be declared to the interviewee)***

My name is ………………………….I am working at Kinshasa School of Public Health, which is a part of University of Kinshasa. I would like to discuss with you and ask you some questions about your community and related health problems. The aim of this interview is to understand how the community is organized in the health zone with regard to health and how different organizations, projects, persons involved in the health sector are working at local level. The information that will be collected will be used to inform the Ministry of Public Health in how to better organize the health provision. I would like to use a voice-recorder during the conversation for better capturing your opinions and would like to have your permission to use it during the discussion. I will show you how it is functioning so you can stop recording when you do not feel comfortable. I will also sometimes take notes in a notebook about the discussion in order to follow up with additional questions.

All discussion will turn around the local community and around health issues. The discussion and your opinion will be kept confidential and will be used only in the purpose of this study. The notes taken during the interview and all materials will be saved in secured places. The recordings will be transcribed verbatim using a computer and afterwards destroyed. During interview, your name or your position will not be mentioned. Some of your personal information will be collected at the end of the interview and written out in the transcript of this interview in a manner to keep anonymous and confidential your identity.

Other persons involved in this project such as supervisors will have access to the transcripts. Additionally, data from this research will be published in review or shared with other persons. In this case, all required strategies will be used in order to ensure the anonymity. All persons involved in the research project are subject to professional ethics and are committed themselves to ethical considerations. Publications that can be produced from the data will all adhere to ethical issues. Your participation is free, you can stop or take decision to not answer to a question without risk for you. You will not receive any direct benefit from participating in this study but information that will be collected will be used to inform better health decision and policy.

You can ask question or make comments on the study and if you have more question or concerns regarding this study and your participation, you can call [Name]………………………………………………….in charge of this research using this phone number…………………………………..or the president of Kinshasa School of Public Health ethics committee.

Name……………………………………………. Phone number………………………………………………………

Do you agree to this? ……………………………………………

You can sign here in order to testify that you were informed and provide you consent:

…………………………………….

Can I begin?

**C. Guide 1. Interview guide for Key informants**

| **N°** | **Questions (Analyse de context)** |
| --- | --- |
|  | **INTRODUCTION**  We would discuss during the interview about the local context of your community. We will discuss about health problems, health organizations and support this community receives. We will also discuss how the community is organized and what its main features in terms of socio-cultural, political, administrative aspects are. |
| 100 | Would you like to present yourself without mentioning your name?  If you are representative of a community group or organization, could you briefly present it? (Probe for goals, missions, activities, achievements, target groups…) |
| 101 | In general, what are health problems population of this community face? Probe for Health issues, health services issues, health related issues?  What are the most concerned people?  Could you provide me what do you think are health needs of this community? |
|  | **HEALTH SUPPORT INTERVENTIONS** |
| 102 | In this health zone, could you provide me what organizations are working in the health sector? Could you link them to health problems you previously mentioned? |
| 102b | You have mentioned ………………………………. What activities do this organization implement in your community or in this health zones? |
| 103 | What are population needs targeted through the implemented activities?  Could you extend on the relevance of the implemented activities?  What results or benefits do you perceive from the implemented activities?  Could you provide some examples? |
|  | **ORGANIZATION OF THE SOCIETY** |
| 104 | What ethnic groups (tribal groups) live in this health zone?  How are this groups organized? Probe for community groups, political groups…)  How do these groups function? (Probe for each type of groups mentioned by the respondent)  How do you think that the existing community groups are involved in the implementation of health activities?  How do you think that the decision making is performed in this group? |
|  | **PLACE DE LA FEMME** |
| 105 | What is the place of women in your community?  What responsibilities are ensured by a woman?  How does woman participate in decision making in the community?  What could you say on community groups gathering women in your community?  What are strengths and weaknesses of this type of groups?  What are health activities that target women?  What are organization that work with women in your community?  What benefits do you think women have in working in community groups? |
|  | **POPULATION AND ORGANIZATIONS** |
| 106 | What organizations, associations or groups could have influence in the health sector or health problems in terms of power, capacity of pressure or community actions?  What sub-groups exist in the community?  How are those groups and sub groups involved in existing health projects and programs?  What role do they play in these projects?  How are they involved?  What interest do you think they have in the activities of other organizations involved in health sector?  How could these groups influence the local community and the health sector? What do you think can be their strengths, weakness or capacity to participate in health sector activities?  What collaboration do you get between existing groups?  What conflicts of interest do you perceived between them?  How do these groups communicate between them and between them and the community? |
| 105 | Could you provide for each of these groups what you think be its influence in existing projects? |
| 106 | What benefits do you think these groups can have from existing projects in health sector? |
| 107 | What contact do these groups have outside the health zone? |
|  | **ACCOUNTABILITY** |
| 108 | How do community group/associations get information about needs, concerns and expectations of their members? For people out of their groups?  How do people who are not members of community association/groups to make their voice heard by associations? |
|  | **POLITIC AND COMMUNITY PARTICIPATION** |
| 109 | What is your opinion about the current policy and political situation with regard to community participation? How does the current political situation influence community engagement and community groups?  What elements from the constitution or other policy do you think support the involvement of community in the management of their community?  What elements from the constitution or other policy do you think support the involvement of community in the management of their health or health services?  What elements do you think prevent community participation?  What elements do you think prevent community participation in the management of their community?  What elements do you think prevent community participation in health and in health services?  What elements do you think prevent social changes in your community? |
| 109a | What community mobilization activities are currently implemented in the community? Could you explain more these activities?  Who do you think take initiative of these activities? What support do organization receive for carrying out these activities (Probe for financial, technical, other…?) |
| **N°** | **INSTITUTIONAL AND LEGAL FRAMEWORK AND GOVERNANCE** |
| 110 | How do you think that political situation affect health sector?  How do you think that political situation affect community groups functioning?  What could you said about decentralization process and about decentralized political and administrative entities? |
| 111 | **FINANCING**  How do you think about socio-economic situation in your area? (Probe for employment, assets, earning potential, occupation, main activities…)  How do this socio-economic situation affect the functioning of local group?  How do this socio-economic situation affect the place of women in your community?  How do this socio-economic situation affect health services and health projects? |
| 112 | **SOCIO-CULTURELS ASPECTS**  Could you provide me some features regarding socio-cultural aspects in your community?  How do you think these features affect community participation?  How do you think these features affect the functioning of community groups?  How the community is informed about what is happened within it? Are there some media that can broadcast community problems? |
| 113 | **COALITION BUILDING**:  How do you think local groups manage the relationship among them?  What platform exist among local groups?  How do the community groups manage their relationship with the health sector at local level? |
| 114 | What relationships do community groups have with administrative and political entities?  What relationships do community groups have with political parties?  What relationships do community groups have with nongovernmental organizations and civil society? |
| 115 | What external actors do you know are interested in the local community?  How do these external actors perceive health projects and interventions with are implemented within the community? |
| 116 | What is your opinion about the organization health projects implemented within the community? |
| 117 | **SOCIAL ACCOUNTABILITY IN HEALTH PROJECTS :**  How do you think health project do for having needs, concerns, demands and expectation of their target groups?  Do you think that health projects know information about needs, concerns, demands and expectations for local community groups? If yes, could you reflect on? |
| 118 | What relationships exist between health projects implemented in the community and their target groups?  What is your opinion on this relationships? |
| 119 | **SATISFACTION AND PERCEPTION :**  As member of this community, how do you think that target groups assess services provided to them through the project? What is your opinion in their satisfaction? |

1. **Identification of respondent Number audio file  :**

***Instructions***: to be filled after the interview

| **Organisation** | **Occupation/Position** | **Sex** | **Age** | **Duration in the position** |
| --- | --- | --- | --- | --- |
|  |  |  |  |  |

QUESTIONNAIRE: FACTORS ASSOCIATED WITH MATERNAL HEALTH SERVICE UPTAKE IN THE MUANDA AND BOLENGE HEALTH ZONES

Questionnaire number: /__/__/__/ Province:

Commune/Territory: Health zone:

Health area: ____________________________

Street/Village: _____________________________ House number _________

Data collector team code: /__/__/

Date: __/…/20…

Team supervisor: ______________________________

SECTION I: CHARACTERISTICS OF PARTICIPANT AND HER HOUSEHOLD

| N° | Questions and filters | | Reponses-codes | | | | | | | | | Codes |
| --- | --- | --- | --- | --- | --- | --- | --- | --- | --- | --- | --- | --- |
| Q100 | Hour of the beginning | | __/__ H __/__ min | | | | | | | | |  |
| Q101 | Who is the head of this household?  *(Instruction : Data collector, tick the sex of the household head)* | Male 1  Female 2 | | | | | | | | | |  |
| Q102 | What is the religion of the household head? (0= No or 1= Yes) | | | | | | | | | | | |
| Q102A | Catholic | 0 | | | 1 | | | | | | |  |
| Q102B | Protestant | 0 | | | 1 | | | | | | |  |
| Q102C | Kimbanguist | 0 | | | 1 | | | | | | |  |
| Q102D | Islam | 0 | | | 1 | | | | | | |  |
| Q102E | Evangelic churches | 0 | | | 1 | | | | | | |  |
| Q102F | Jehovah Witness | 0 | | | 1 | | | | | | |  |
| Q102G | Other Christian churches | 0 | | | 1 | | | | | | |  |
| Q102H | Animist | 0 | | | 1 | | | | | | |  |
| Q102I | Agnostic/Atheist | 0 | | | 1 | | | | | | |  |
| Q102J | Other (to be specified….) | 0 | | | 1 | | | | | | |  |
| Q103 | How old is the head of this household?(years) | ____ | | | | | | | | | |  |
| Q104 | What is his/her main occupation? (Tick 0=No or 1=Yes) | | | | | | | | | | |  |
| Q104A | Civil servant/Military/policeman | | | 0 | | | | | | 1 | |  |
| Q104B | Employed in private sector |  | | 0 | | | | | | 1 | |  |
| Q104C | Farmer/ Fisheries and livestock |  | | 0 | | | | | | 1 | |  |
| Q104D | Traders/Small traders |  | | 0 | | | | | | 1 | |  |
| Q104E | Odd jobs |  | | 0 | | | | | | 1 | |  |
| Q104F | No formal occupation |  | | 0 | | | | | | 1 | |  |
| Q104G | Other (to be specified…………) | | | 0 | | | | | | 1 | |  |
| Q105 | What is the highest education level the head of this household had completed? | | | | | | | | | | |  |
| Q105A | No completed primary school |  | | 0 | | | | | | 1 | |  |
| Q105B | Completed primary school |  | | 0 | | | | | | 1 | |  |
| Q105C | No completed secondary school |  | | 0 | | | | | | 1 | |  |
| Q105D | Completed secondary school |  | | 0 | | | | | | 1 | |  |
| Q105E | No completed High school |  | | 0 | | | | | | 1 | |  |
| Q105F | Completed High school and above | | | 0 | | | | | | 1 | |  |
| Q106 | Is your partner the head of the household? | No 0  Yes 1 | | | | | | | | | | 1→107 |
| Q106A | If Q106=0, what is the main occupation of your partner? (Tick 0=No or 1=Yes) | | | | | | | | | | |  |
| Q106A | Civil servant/military/policeman | | | 0 | | | | | | 1 | |  |
| Q106B | Employed in private sector |  | | 0 | | | | | | 1 | |  |
| Q106C | Farm/Fisheries/Livestock |  | | 0 | | | | | | 1 | |  |
| Q106D | Traders/small traders |  | | 0 | | | | | | 1 | |  |
| Q106E | Odd jobs |  | | 0 | | | | | | 1 | |  |
| Q106F | No formal occupation |  | | 0 | | | | | | 1 | |  |
| Q106G | Other (to be précised……) | | | 0 | | | | | | 1 | |  |
| Q106H | How old is he? | ____ | | | | | | | | | |  |
| Q106I | What is the highest education level your partner had completed? | | | | | | | | | | |  |
| Q106J | No completed primary school | 0 | | | | | | | 1 | | |  |
| Q106K | Completed primary school | 0 | | | | | | | 1 | | |  |
| Q106M | No completed secondary school | 0 | | | | | | | 1 | | |  |
| Q106N | Completed secondary school | 0 | | | | | | | 1 | | |  |
| Q106O | No completed High school | 0 | | | | | | | 1 | | |  |
| Q106P | Completed High school and above | 0 | | | | | | | 1 | | |  |
| Q107 | How many people live in the same house than you including yourself? | ____ | | | | | | | | | |  |
| Q108 | How much do you spend every day for your household? | __________________FC | | | | | | | | | |  |
| Q109 | How much does the head of this household earn every month including salaries, premium and other resources? | __________________FC | | | | | | | | | |  |
| **Information on the participant (woman aged 15-49 expectant of more than 3 months or ever delivered for less than 6 months)** | | | | | | | | | | | | |
| Q110 | How old are you? (year) | ____ | | | | | | | | | |  |
| Q111 | What is your ethnic group? |  | | | | | | | | | |  |
| Q112 | What is the highest education level had you completed? | | | | | | | | | | |  |
| Q112A | No completed primary school | 0 | | | | 1 | | | | | |  |
| Q112B | Completed primary school | 0 | | | | 1 | | | | | |  |
| Q112C | No completed secondary school | 0 | | | | 1 | | | | | |  |
| Q112D | Completed secondary school | 0 | | | | 1 | | | | | |  |
| Q112E | No completed High school | 0 | | | | 1 | | | | | |  |
| Q112G | Completed High school and above | 0 | | | | 1 | | | | | |  |
| Q113 | Are you married? | Single 1  Married monogamous 2  Married polygamous 3  Live in partnership 4  Divorced /separated 5  Widow 6 | | | | | | | | | |  |
| Q114 | What is your main occupation? (Tick 0=No or 1=Yes) | | | | | | | | | | |  |
| Q114A | Civil service/military/police | | |  | | | | | | |  |  |
| Q114B | Private sector |  | | 0 | | | | | | | 1 |  |
| Q114C | Farm/fisheries/ |  | | 0 | | | | | | | 1 |  |
| Q114D | Trade/small trade |  | | 0 | | | | | | | 1 |  |
| Q114E | Odd jobs |  | | 0 | | | | | | | 1 |  |
| Q114F | No occupation |  | | 0 | | | | | | | 1 |  |
| Q114G | Other (to be precised……) | | | 0 | | | | | | | 1 |  |
| Q115 | What is your religion? | | | | | | | | | | |  |
| Q115A | Catholic | 0 | | | | | 1 | | | | |  |
| Q115B | Protestant | 0 | | | | | 1 | | | | |  |
| Q115C | Kimbanguist | 0 | | | | | 1 | | | | |  |
| Q115D | Islam | 0 | | | | | 1 | | | | |  |
| Q115E | Evangelic churches | 0 | | | | | 1 | | | | |  |
| Q115F | Jehovah Witnesses | 0 | | | | | 1 | | | | |  |
| Q115G | Other Christian churches | 0 | | | | | 1 | | | | |  |
| Q115H | Animist | 0 | | | | | | 1 | | | |  |
| Q115I | Agnostic/atheist | 0 | | | | | | 1 | | | |  |
| Q115J | Other (to be specified….) | 0 | | | | | | 1 | | | |  |
| Q116 | How often have you ever given birth (live birth or not)? | ____ | | | | | | | | | |  |
| Q117 | How do you do for going to the local health facility? | On foot 1  Bicycle 2  Motorcycle 3  Car 4  Boat 5 | | | | | | | | | |  |
| Q118 | How many kilometers is your home to the local health facility? | ____ km | | | | | | | | | |  |
| Q119 | How much time do you take to reach the local health facility? | ____ | | | | | | | | | |  |

SECTION II: UPTAKE, PERCEPTION AND ASSESSMENT OF HEALTH SERVICE QUALITY

| ***Instructions : Q201, Q202 et Q203 are to be asked to expectant and***  ***Q204 et Q205C to ever delivered*** | | | | | | | |
| --- | --- | --- | --- | --- | --- | --- | --- |
| Q200 | Check the participant, is she expectant? | No 0  Yes 1 | | | | | 0→ Q204 |
| Q201 | If expectant, have you already attended for antenatal care? | No 0  Yes 1 | | | | |  |
| Q202 | How many sessions have already you attended? | ____ | | | | |  |
| Q203 | Where do you attend the antenatal care? | The local health centre 1  Health post in the health area 2  Private facility in the health area 3  A health facility out of the health area 4  Other (to be specified)…………10 | | | | | 1,2,3,4,10→ Q206 |
| Q204 | Check is the respondent is an ever delivered? | No 0  Yes 1 | | | | |  |
| Q204B | How many months do you spend since you gave birth? | ____ | | | | |  |
| Q205A | Where did you give birth? | The local health centre 1  Health post in the health area 2  Private facility in the health area 3  A health facility out of the health area 4  At home 5  Other (to be specified)…………10 | | | | |  |
| Q205B | During your last pregnancy, did you attend antenatal care? | No 0  Yes 1 | | | | | 0→ Q206 |
| Q205C | How many antenatal care sessions did you attend in total? | ____ | | | | |  |
| Dear participant,  I will recount to you two short stories about two ladies living in another village. The first story is about a lady named Marie. This lady was seven months' pregnant and went to the local health centre for antenatal care. Although she reached the health centre on time, the nurse made her wait on a bench for more than four hours before attending to her. While attending to her, the nurse left the door open, allowing other patients to see her body and Marie was asked for extra money at the end of the examination.  I would like to know whether you encountered a similar situation yourself during your last visit to the local health centre for antenatal care. Have you ever encountered another situation that you were unhappy with?  The second story is about another lady named Anne who, for her second pregnancy, visited the local health centre for the delivery. As she did not appropriately follow the antenatal care schedule, the midwife scolded her in front of other women and did not assist her when her labour started, and slapped her on the thigh because she was unable to push the baby out.  I would like to know whether you encountered a similar situation yourself during your last visit to the local health centre for delivery. Have you ever encountered another situation that you were unhappy with? | | | | | | | |
| Q206 | Have you ever encountered another situation that you were unhappy with? | No 0  Yes 1 | | | | |  |
| Q207 | ***Instructions : Data collector, monitor by checking in this list probable disrespectful event during health service attendance:***  I would like to know if you were confronted to the following event during your visit in the health centre. (Tick 0=No or 1=Yes) | | | | | | |
| Q207A | No respect of the dignity of the person: | A.1 Shout at the patient | | | 0 | 1 |  |
|  |  | A.2 Threaten to withhold treatment | | | 0 | 1 |  |
|  |  | A.3 Threatening or negative comments | | | 0 | 1 |  |
| Q207B | Abandonment : | B.1 Ignored or abandoned when asking for need | | | 0 | 1 |  |
|  |  | B.2 Delivered alone/  No performance of some healthcare during antenatal care visit | | | 0 | 1 |  |
| Q207C | Physical abuse | C.1 Hit | | | 0 | 1 |  |
|  |  | C.2 Beat | | | 0 | 1 |  |
|  |  | C.3 Slap | | | 0 | 1 |  |
|  |  | C.4 Push | | | 0 | 1 |  |
|  |  | C.5 Pinch | | | 0 | 1 |  |
|  |  | C.6 Sexual abuse | | | 0 | 1 |  |
|  |  | C.7 Otherwise hurt | | | 0 | 1 |  |
| Q207D | No confidentiality : | D.1 Allow your body to be seen by other | | | 0 | 1 |  |
|  |  | D.2 Divulgate confidential information | | | 0 | 1 |  |
| Q207E | Non consent care : | E.1 Carry out healthcare without explanation of permission | | | 0 | 1 |  |
| Q207F | Inappropriate demand of payment | F.1 Demand of informal payment | | | 0 | 1 |  |
|  |  | F.2 Detention of mother or new-born at the health facility due to failure to payment | | | 0 | 1 |  |
| Q207G | Attention : | G.1 Prompt response when required | | | 0 | 1 |  |
| Q207H | Environment | H.1 Clean rooms in the health facility | | | 0 | 1 |  |
|  |  | H.2 Small room in the health facility | | | 0 | 1 |  |
| Q207 I | Choice of the health provider | I.1 Have you chosen the health provider to attend for? | | | 0 | 1 |  |
| Q208 | Have you ever learned similar event from your relatives or neighbour? | No 0  Yes 1 | | | | |  |
| Q209 | ***(Instruction*** ***: Q209 to 214E if respondent answered yes to previous question)***  Have you expressed your concerns or complains to the concerned health provider? | No 0  Yes 1 | | | | |  |
| Q210 | Why? (for yes or no) |  | | | | |  |
| Q211 | If yes, did the health provider change his behaviour or apologize? | No 0  Yes 1 | | | | |  |
| Q212 | What motivate you to express yourself your concerns to health providers? |  | | | | |  |
| Q213 | Apart from the health providers, have you shared your concerns with another person? | No 0  Yes 1 | | | | |  |
| Q214 | If yes, could you mention me with whom you shared your concerns (Tick the code corresponding to the mentioned person (Circle 0=No or 1=Yes) | | | | | | |
| Q214A | Husband | 0 | 1 | Why? | | |  |
| Q214B | Parent (Mother, Aunt, sister) | 0 | 1 |  | | |  |
| Q214C | Community health workers | 0 | 1 |  | | |  |
| Q214D | Other community leaders or authorities | 0 | 1 |  | | |  |
| Q214E | The nurse in-charge | 0 | 1 |  | | |  |
| Q215 | Based on your experience, how do you assess the quality of care in this health facility? (If it is asked to you to grade out of five) | 5=Very satisfied 1  4=Satisfied 2  3= Neutral 3  2=Unsatisfied 4  1=No at all satisfied 5 | | | | |  |

SECTION III: PERCEPTION ON CLIENT CONSIDERATION AND COMMUNICATION

| Q301 | During the visit, did the health provider explain you your health problem? | No 0  Yes 1 |  |
| --- | --- | --- | --- |
| Q302 | Did the health provider let express or ask you your view about the choice of the treatment? | No 0  Yes 1 |  |
| Q303 | Have you been informed of issues of the health facility before to visit (schedule, care, providers, rules, other)? | No 0  Yes 1 |  |
| Q304 | If yes, how have you been informed of them? | Community health worker 1 Health committee member 2  Health providers 3 Relative/Neighbours 4  Other (to specify)……………10 |  |
| Q305 | Is the health provider or the health facility collect patient’s view or opinions as users? | No 0  Yes 1 | 0→ Q401 |
| Q306 | Si yes, what ways are used | Suggestion box 1  Concern form 2  Face-to-face interview 3  Meeting with community leaders 4  Informal discussion with client/community 5  Direct feedback 6  Other (to specify)……………10 |  |
| Q307 | Do you think that health providers take into account your opinion? | No 0  Yes 1 |  |
| Q308 | How do you know that they are taking into account your view? | Improvement of the reception 1  Moral assistance by providers 2  Good collaboration with provider after feedback 3  Application of recommendation or suggestion 4  Explanation of health service 5 |  |

SECTION IV: INTENTION TO FUTURE VISIT OR TO RECOMMEND RELATIVES BASED ON RECENT EXPERIENCE

| Q401 | Have you encountered any complications during your pregnancy or delivery? | No 0  Yes 1 | | |  |
| --- | --- | --- | --- | --- | --- |
| Q402 | If depending on you, would you like to visit again the same health facility in future or recommend your relative for antenatal care or delivery? | No 0  Yes 1 | | |  |
| Q403 | Could you explain why? | | | |  |
| Q403A | Good reception | | 0 | 1 |  |
| Q403B | Good collaboration with health providers | | 0 | 1 |  |
| Q403C | Good process in follow up of pregnancy | | 0 | 1 |  |
| Q403D | Good management of the new born | | 0 | 1 |  |
| Q403E | Good quality of care | | 0 | 1 |  |
| Q403F | Use to attend the health facility | | 0 | 1 |  |
| Q403G | Affordable healthcare cost | | 0 | 1 |  |
| Q403H | Clean sanitation | | 0 | 1 |  |
| Q403I | Near to my home | | 0 | 1 |  |
| Q403J | Fair health providers | | 0 | 1 |  |
| Q403K | Insufficient financial resources | | 0 | 1 |  |
| Q403L | Skilled health providers | | 0 | 1 |  |
| Q403M | Health providers behave finely with patient | | 0 | 1 |  |
| Q403N | No clear reason | | 0 | 1 |  |
| Q403N | Preference for home delivery | | 0 | 1 |  |
| Q404 | Hour (end of the survey) | __/__ hour__/__ min | | |  |

Thank you for your participation. Have you a question?
